# Diterpene Glycosides from *Stevia rebaudiana*

**DOI:** 10.3390/molecules16053552

**Published:** 2011-04-28

**Authors:** Venkata Sai Prakash Chaturvedula, Mani Upreti, Indra Prakash

**Affiliations:** The Coca-Cola Company, Organic Chemistry Department, Research and Technology, One Coca-Cola Plaza, Atlanta, GA 30313, USA

**Keywords:** *Stevia rebaudiana*, Compositae, Asteraceae, diterpenoid glycosides, spectral data, chemical studies

## Abstract

Three novel diterpene glycosides were isolated for the first time from the commercial extract of the leaves of *Stevia rebaudiana*, along with several known steviol glycosides, namely stevioside, rebaudiosides A-F, rubusoside and dulcoside A. The new compounds were identified as 13-[(2-*O*-β-d-glucopyranosyl-3-*O*-β-d-glucopyranosyl-β-d-glucopyranosyl)oxy] *ent*-kaur-15-en-19-oic acid (**1**), 13-[(2-*O*-β-d-glucopyranosyl-3-*O*-β-d-glucopyranosyl-β-d-glucopyranosyl)oxy]-16β-hydroxy-*ent*-kauran-19-oic acid (**2**) and 13-methyl-16-oxo-17-nor-*ent*-kauran-19-oic acid-β-d-glucopyranosyl ester (**3**) on the basis of extensive 2D NMR and MS spectroscopic data as well as chemical studies.

## 1. Introduction

*Stevia rebaudiana* (Bertoni) Bertoni is a perennial shrub belonging to the family of Asteraceae (Compositae) native to Brazil and Paraguay, but now grown commercially in a number of countries, particularly in Japan, Taiwan, Korea, Thailand and Indonesia [1-2]. Extracts of the leaves of *S. rebaudiana* have been used for decades to sweeten food and beverages in Japan, South America and China. The major constituents in the leaves of *S. rebaudiana* are the potently sweet diterpenoid glycosides stevioside, rebaudiosides A and D, and dulcoside A. These compounds, which are known as Stevia sweeteners, are the glycosides of the diterpene steviol, *ent*-13-hydroxykaur-16-en-19-oic acid [3]. As a part of our continuing research to discover natural sweeteners [4-5], we have collected commercial extracts of *S. rebaudiana* from various suppliers all over the World. This paper describes the isolation of three diterpene glycosides **1**-**3** isolated for the first time from the crude extract of the leaves of *S. rebaudiana* obtained from ShenZhen NII Natural Food Ingredient Co. Ltd, China and their identification based on extensive spectroscopic (NMR and MS) and chemical studies.

## 2. Results and Discussion

**Figure 1:**
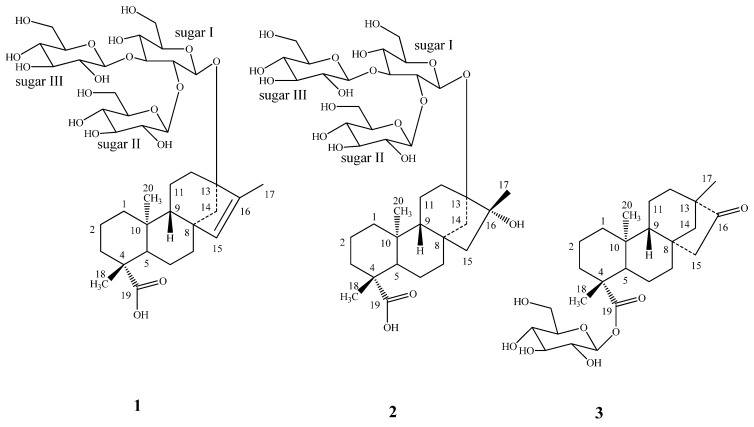
Structures of **1**-**3**.

Purification of the commercial extract of *S. rebaudiana* obtained as Lot No: 20071003 from ShenZhen NII Natural Food Ingredient Co. Ltd, China, resulted in the isolation of three new diterpenoid glycosides **1**-**3**, and the known steviol glycosides, stevioside, rebaudiosides A-F, rubusoside and dulcoside A (Figure 1). The structures of the known compounds were identified in comparison of their retention times with authentic standards using the HPLC-MS method as described previously [6] and the spectral data that were reported in the literature [7,8,9,10,11,12,13]. 

**Figure 2:**
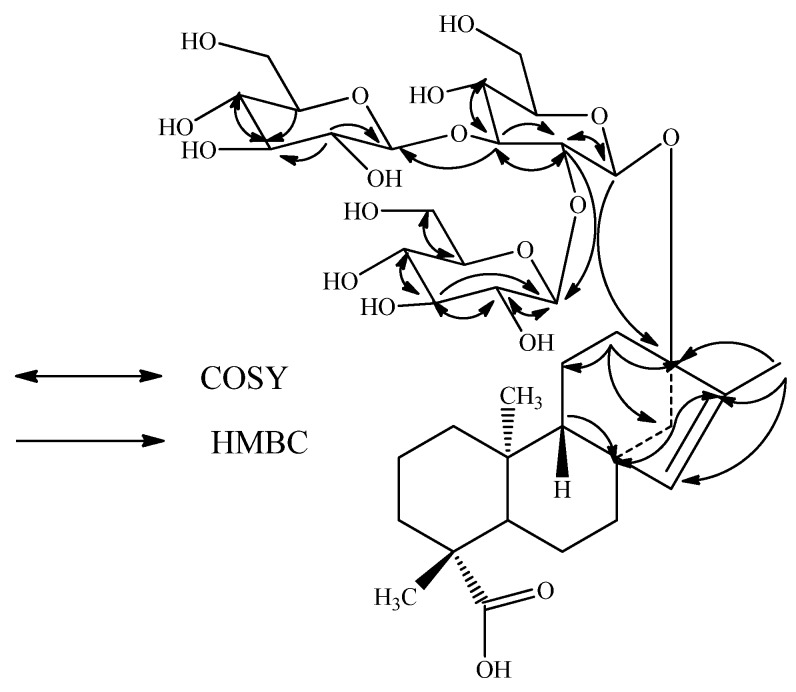
Key COSY and HMBC correlations of **1**.

Compound **1** was obtained as a white powder and its molecular formula was assigned as C_38_H_60_O_18_ from its HRESI mass spectrum, which showed a (M + Na)^+^ ion at *m/z* 827.3661; this was supported by the ^13^C-NMR spectral data. The ^1^H-NMR spectrum of **1** (Table 1) showed the presence of three methyl singlets at δ 0.99, 1.17, and 1.72, eight methylene and two methine protons between δ 0.86-2.23, and a trisubstituted olefinic proton at δ 5.14, similar to *ent*-13-hydroxykaur-15-en-19-oic acid [15]. The basic skeleton of kaurane diterpenoids was supported by COSY (H-1/H-2; H-2/H-3; H-5/H-6; H-6/H-7; H-9/H-11; H-11/H-12) and HMBC (H-1/C-2, C-10; H-3/C-1, C-2, C-4, C-5, C-18, C-19; H-5/C-4, C-6, C-7, C-9, C-10, C-18, C-19, C-20; H-9/C-8, C-10, C-11, C-12; H-14/C-8, C-9, C-13, C-15, C-16 and H-17/C-13, C-15, C-16) correlations (Figure 2). In addition, the ^1^H-NMR spectrum of **1** also showed three anomeric protons as doublets at δ 4.66 (*J* = 7.8 Hz), 4.85 (*J* = 7.8 Hz), and 4.82 (*J* = 7.8 Hz) suggesting the presence in its structure of three sugar units, which was supported by the fragment ions observed at *m/z* 643, 481, and 319 in the ESI MS/MS spectrum corresponding to the successive loss of three hexose moieties from its [M + H]^+^ ion. Enzymatic hydrolysis of **1** furnished a compound which was found identical to *ent*-13-hydroxykaur-15-en-19-oic acid on the basis of NMR spectral data [13-14]. Acid hydrolysis of **1** afforded d-glucose that was identified by preparing the corresponding thiocarbamoyl-thiazolidine carboxylate derivative with L-cysteine methyl ester and *O*-tolyl isothiocyanate, and in comparison of its retention time with the standard sugars as described in the literature comparison [15].

The ^1^H- and ^13^C-NMR values for all the protons and carbons were assigned on the basis of COSY, HSQC and HMBC correlations (Figure 2) and are given in Table 1 and Table 2. A close comparison of the ^1^H- and ^13^C-NMR values of **1** with those of rebaudioside A suggested a 2,3-branched β-d-glucotriosyl substituent at C-13 as well as the absence of a glucosyl unit at C-19 and migration of the exocyclic double bond from C-16/C-17 to C-15/C-16. This was supported by the ^13^C-NMR values for the trisubstituted double bond between C-15 and C-16 which were observed at δ 136.1 and 142.6, respectively, and also from the HMBC correlations: H-12/C-9, C-11, C-13, C-14, C-16; H-15/C-8, C-9, C-14, C-16, C-17 and H-17/C-13, C-15, C-16. The large coupling constants observed for the three d-glucose anomeric protons suggested the β-orientation as reported for steviol glycosides [6,7,8,9,10,11,12,13]. Thus, structure of **1** was established as 13-[(2-*O*-β-d-glucopyranosyl-3-*O*-β-d-glucopyranosyl-β-d-glucopyranosyl)oxy] *ent*-kaur-15-en-19-oic acid.

**Figure 3:**
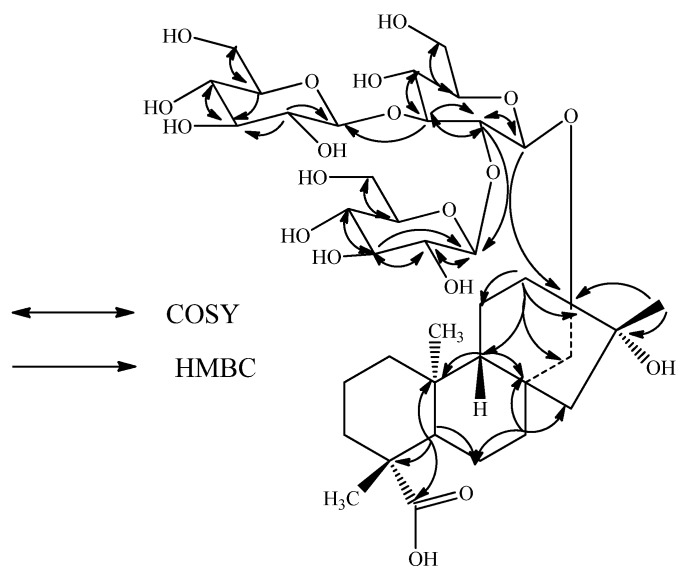
Key COSY and HMBC correlation of **2**.

The molecular formula of compound **2** was determined to be C_38_H_62_O_19_ from the [M + H]^+^ ion at *m/z* 823, together with an [M + Na]^+^ adduct at *m/z* 845 in the positive ESI mass spectrum, which was confirmed by the HRMS data. The ^1^H-NMR spectrum of **2** (Table 1) showed the presence of three methyl singlets, nine methylene and two methine protons. In the absence of any unsaturated protons together with the appearance of a methyl group at δ 1.27 indicated that the structure of **2** should be similar to *ent*-13,16-dihydroxykauran-19-oic acid [13-14] which was supported by the δ_C_ 76.8 corresponding to the tertiary hydroxyl at C-16. The presence of *ent*-13,16-dihydroxykaurane skeleton in **2** was supported by the key HMBC correlations: H-12/C-11, C-13, C-14, C-16; H-14/C-8, C-12, C-13, C-15, C-16; H-17/C-13, C-15, C-16 (Figure 3). The ESI MS/MS spectrum of **2** showed the fragment ions at *m/z* 661, 499, and 337 corresponding to the successive loss of three hexose moieties from its [M + H]^+^ ion and this was supported by the three anomeric protons observed at δ 4.66, 4.74 and 4.89 in its ^1^H-NMR spectral data. Acid hydrolysis of **2** performed as in **1** afforded d-glucose confirming the three sugar molecules present in **2** as glucosyl units. The ^1^H- and ^13^C-NMR values for all the protons and carbons were assigned on the basis of COSY, HMQC and HMBC correlations (Table 1 and Table 2). 

Enzymatic hydrolysis of **2** furnished a compound which was found identical to *ent*-13, 16β-dihydroxykauran-19-oic acid on the basis of NMR spectral data comparisons [13-14]. A close comparison of the ^1^H- and ^13^C-NMR values of **2** with those of **1** suggested the presence of three glucose units attached as a 2,3-branched β-d-glucotriosyl substituent at C-13 hydroxyl of *ent*-13, 16β-dihydroxykauran-19-oic acid. The large coupling constants observed for the three anomeric protons of the glucose moieties at δ 4.66 (d, *J* = 8.2 Hz), 4.74 (d, *J* = 7.8 Hz), and 4.89 (d, *J* = 7.8 Hz), suggested the β-orientation as in **1**. Considering the stereochemistry for the 16-hydroxyl group in **2** as β on the basis of the acid obtained by enzymatic hydrolysis, the structure was thus deduced to be13-[(2-*O*-β-d-glucopyranosyl-3-*O*-β-d-glucopyranosyl-β-d-glucopyranosyl)oxy]-16β-hydroxy *ent*-kauran-19-oic acid. 

**Figure 4:**
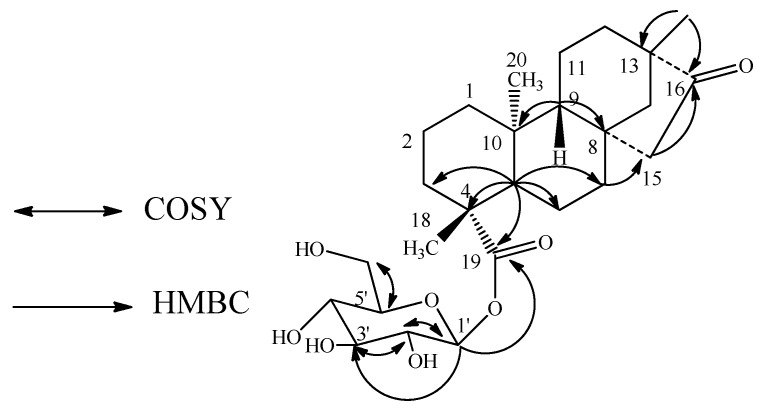
Key COSY and HMBC correlations of **3**.

The molecular formula of compound **3** was deduced as C_26_H_40_O_8_ from the [M + H]^+^ ion observed at *m/z* 481, together with an [M + Na]^+^ adduct at *m/z* 503 in the positive ESI mass spectrum and this was supported by the HRMS data. The ^1^H-NMR spectrum of **3** (Table 1) also showed the presence of three methyl singlets at δ 0.81, 0.94 and 1.23; nine methylene and two methine protons, similar to **2**. Enzymatic hydrolysis of **3** furnished a compound which was found identical to isosteviol on the basis of its NMR spectral data, identical to the reported values in the literature [14]. The presence of isosteviol skeleton was supported by the key HMBC correlations: H-12/C-9, C-11, C-13, C-14, C-16, C-17; H-14/C-8, C-12, C-13, C-15, C-16, C-17; H-15/C-8, C-14, C-16; H-17/C-13, C-14, C-16. The ^1^H NMR spectrum also showed an anomeric proton as doublet at δ 5.39 (*J* = 8.2 Hz), suggesting the presence of one sugar residue in its structure. Acid hydrolysis of **3** afforded d-glucose, confirming the sugar unit present in **3** as glucose as in **1** and **2**. The placement of the glucose moiety in **3** was assigned to be at C-19 based on the key COSY and HMBC correlations shown in Figure 4. The ^1^H and ^13^C NMR values for all the protons and carbons were assigned on the basis of COSY, HMQC and HMBC correlations (Table 1 and Table 2). Based on the results from chemical and spectral studies, **3** was assigned as 13-methyl-16-oxo-17-nor-*ent*-kauran-19-oic acid-β-d-glucopyranosyl ester.

This is the first report of the occurrence of the diterpene glycosides **1**-**3** from *S. rebaudiana* in nature, which were reported earlier as rebaudioside A derivative/degradation products [16-17]. Also, this is the first report of their complete ^1^H- and ^13^C-NMR spectral assignments that were made on the basis of spectral (COSY, HSQC, HMBC, and MS/MS) and chemical studies. 

## 3. Experimental 

### 3.1. General

Melting points were measured using a SRS Optimelt MPA 100 instrument and are uncorrected. Optical rotations were recorded using a Rudolph Autopol V at 25 °C and NMR spectra were acquired on Bruker Avance DRX 500 MHz and Varian Unity Plus 600 MHz instruments using standard pulse sequences. The spectra were referenced to the residual solvent signal (δ_H_ 3.30, δ_C_ 49.0 for CD_3_OD), chemical shifts are given in δ (ppm), and coupling constants are reported in Hz. MS and MS/MS data were generated with a Waters Premier Quadrupole Time-of-Flight (Q-Tof) mass spectrometer equipped with an electrospray ionization source operated in the positive-ion mode and Thermo Fisher Discovery OrbiTrap in the positive mode electrospray. Samples were diluted with water: acetonitrile (1:1) containing 0.1% formic acid and introduced via infusion using the onboard syringe pump. Preparative HPLC was performed on an Agilent 1100 system using a Phenomenex Prodigy ODS (3) column (250 × 21.2 mm, 5 μm).

### 3.2. Plant Material

A commercial Stevia extract with Lot No.: 20071003 was obtained from ShenZhen NII Natural Food Ingredient Co. Ltd, China. A voucher specimen is deposited at The Coca-Cola Company, No. VSPC-3166-153.

### 3.3. Isolation

Preliminary separation of the crude extract from the leaves of *S. rebaudiana* SRA 40, Lot No. 20071003 consisting of a mixture of various diterpenoid glycosides including 40-60% rebaudioside A was carried out via preparative HPLC on an Agilent 1100 system. The HPLC method A involved using Phenomenex Prodigy Column C_18_ (250 × 10 mm, 10 μm); UV Detection: 220 nm; Mobile Phase A: H_2_O (0.1% TFA); Mobile Phase B: Acetonitrile; Flow Rate: 5.0 mL/min; Injection volume: 400 μL. The gradient increased from 95: 5 (A: B) to 0: 100 (A: B) over 30 min, and remained at 0: 100 (A: B) for 5 min. Removal of the dominant rebaudioside A (*t_R_* 17.13 min) using the above HPLC method and subsequent collection of the peak eluting at *t_R_* 14.60 min over multiple runs furnished **1** (4.6 mg). The peak eluting at *t_R_* 12.86 min contained a mixture of **2** along with several other compounds. Since **2** was only partially resolved under the first HPLC method, a second round of purification was applied to isolate the pure compound. This was carried out using HPLC method B which was a modification to HPLC method A using the gradient of 80: 20 (A: B) to 70: 30 (A: B) applied for 40 min. The peak eluted at *t_R_* 32.4 min was confirmed to correspond to **2** (3 mg). Similar to **1**, removal of rebaudioside A and combining the peak eluted at *t_R_* 18.2 min using HPLC method A over multiple runs and concentration under vacuum furnished **3** (4.2 mg). All the known compounds were identified using HPLC-MS in comparison with authentic standards as described previously [6] and the spectral data that were reported in the literature [7,8,9,10,11,12,13].

*13-[(2-O-β-d-glucopyranosyl-3-O-β-d-glucopyranosyl-β-d-glucopyranosyl)oxy] ent-kaur-15-en-19-oic acid* (**1**). White powder, mp 254.5-254.7 °C, [α]_D_^25^ -49.56 (*c* 1.0, MeOH); ^1^H-NMR (500 MHz, CD_3_OD, δ ppm) and ^13^C-NMR (125 MHz, CD_3_OD, δ ppm) spectroscopic data see Table 1 and Table 2; HRMS (M + Na)^+^ *m/z* 827.3661 (calcd. for C_38_H_60_O_18_Na: 827.3677)

*Enzymatic hydrolysis of* **1**. A solution of **1** (250 μg) was dissolved in 0.1 M sodium acetate buffer, pH 4.5 (2.5 mL) and crude pectinase from *Aspergillus niger* (50 μL, Sigma-Aldrich, P2736) was added. The mixture was stirred at 50 °C for 48 hr. The product precipitated out during the reaction and was filtered and then crystallized from methanol (MeOH). The resulting product was identical to *ent*-13-hydroxykaur-15-en-19-oic acid by comparison of their ^1^H-NMR spectral data [13-14].

*Acid Hydrolysis of* **1**. Compound **1** (500 μg) was hydrolyzed with 0.5 M HCl (0.5 mL) for 1.5 h. After cooling, the mixture was passed through an Amberlite IRA400 column and the eluate was lyophilized. The residue was dissolved in pyridine (0.25 mL) and heated with L-cysteine methyl ester HCl (2.5 mg) at 60 °C for 1.5 h, and then *O*-tolyl isothiocyanate (12.5 μL) was added to the mixture and heated at 60 °C for an additional 1.5 h. The reaction mixture was analyzed by HPLC: column Phenomenex Luna C18, 150 × 4.6 mm (5 u); 25% acetonitrile-0.2% TFA water, 1 mL/min; UV detection at 250 nm. The sugar was identified as d-glucose (*t*R, 12.26 min) [authentic samples, d-glucose (*t*R, 12.35) and L-glucose (*t*R, 11.12 min)] [15].

*13-[(2-O-β-d-glucopyranosyl-3-O-β-d-glucopyranosyl-β-d-glucopyranosyl)oxy]-16β-hydroxy-ent-kauran-19-oic acid* (**2**). White powder, mp 220.4-221.2 °C, [α]_D_^25^ -32.60 (*c* 1.0, MeOH); ^1^H-NMR (500 MHz, CD_3_OD, δ ppm) and ^13^C-NMR (125 MHz, CD_3_OD, δ ppm) spectroscopic data see Table 1 and Table 2; HRMS (M + Na)^+^ *m/z* 845.3765 (calcd. for C_38_H_62_O_19_Na: 845.3783)

*Enzymatic hydrolysis of **2***. Enzymatic hydrolysis of **2** (500 μg) as described above furnished *ent*-13, 16β-dihydroxykauran-19-oic acid which was confirmed on the basis of ^1^H NMR spectral data [13,14]. 

*Acid Hydrolysis of **2***. Hydrolysis of **2** (500 μg) as described above furnished d-glucose [15].

*13-methyl-16-oxo-17-nor-ent-kauran-19-oic acid-β-d-glucopyranosyl ester* (**3**). White powder, mp 172.5 °C, [α]_D_^25^ -55.83 (*c* 1.0, EtOH); ^1^H-NMR (500 MHz, CD_3_OD, δ ppm) and ^13^C-NMR (125 MHz, CD_3_OD, δ ppm) spectroscopic data see Table 1 and Table 2; HRMS (M + Na)^+^ *m/z* 503.2608 (calcd. for C_26_H_40_O_8_Na: 503.2621)

*Enzymatic hydrolysis of* **3**. Enzymatic hydrolysis of **3** (500 μg) as described above furnished isosteviol which was confirmed on the basis of ^1^H-NMR spectral data [14]. 

*Acid Hydrolysis of* **3**. Hydrolysis of **2** (500 μg) as described above furnished d-glucose [15].

## 4. Conclusions

Three diterpenoid glycosides **1**-**3** were isolated from a commercial extract of the leaves of *S. rebaudiana* obtained from ShenZhen NII Natural Food Ingredient Co. Ltd, China, along with the known steviol glycosides stevioside, rebaudiosides A-F, rubusoside and dulcoside A. The new compounds were identified as 13-[(2-*O*-β-d-glucopyranosyl-3-*O*-β-d-glucopyranosyl-β-d-gluco-pyranosyl)oxy] *ent*-kaur-15-en-19-oic acid, 13-[(2-*O*-β-d-glucopyranosyl-3-*O*-β-d-glucopyranosyl-β-d-glucopyranosyl)oxy]-16β-hydroxy-*ent*-kauran-19-oic acid, 13-methyl-16-oxo-17-nor-*ent*-kauran-19-oic acid-β-d-glucopyranosyl ester on the basis of spectroscopic and chemical studies. This is the first report of the isolation of these three diterpene glycosides from *S. rebaudiana* in nature. The discovery of these compounds is an important addition in expanding our understanding of the diversity of the diterpenoid glycosides present in the *S. rebaudiana*.

## Figures and Tables

**Table 1:** ^1^H-NMR chemical shift values for **1**–**3** isolated from *Stevia rebaudiana* recorded in CD_3_OD ^a^.

Position	1	2	3
1	0.86 (m, 1H), 1.86 (m, 1H)	0.86 (m, 1H), 1.86 (m, 1H)	0.97 (m, 1H), 1.71 (m, 1H)
2	1.39 (m, 1H), 1.90 (m, 1H)	1.42 (m, 1H), 1.94 (m, 1H)	1.38 (m, 1H), 1.90 (m, 1H)
3	1.02 (m, 1H), 2.10 (d, 11.9, 1H)	1.00 (m, 1H), 2.12 (d, 12.6, 1H)	1.08 (m, 1H), 2.18 (d, 13.7, 1H)
5	1.08 (m, 1H)	1.07 (m, 1H)	1.22 (m, 1H)
6	1.52 (m, 1H), 1.83 (m, 1H)	1.82 (m, 1H), 1.97 (m, 1H)	1.90 (m, 2H)
7	1.50 (m, 1H), 1.58 (m, 1H)	1.37 (m, 1H), 1.58 (m, 1H)	1.49 (m, 1H), 1.68 (m, 1H)
9	0.86 (m, 1H)	0.93 (t, *J* = 7.8 Hz, 1H)	1.24 (m, 1H)
11	1.50 (m, 1H), 1.69 (m, 1H)	1.65 (m, 1H), 1.85 (m, 1H)	1.23 (m, 1H), 1.68 (m, 1H)
12	1.63 (m, 1H), 1.67 (m, 1H)	1.80 (m, 1H), 1.04 (m, 1H)	1.43 (m, 1H), 1.53 (m, 1H)
14	1.68 (m, 1H), 2.23 (d, 10.0, 1H)	1.84 (m, 1H), 1.98 (d, 10.0, 1H)	1.45 (m, 1H), 1.56 (m, 1H)
15	5.14 (s, 1H)	1.42 (d, 13.7, 1H), 1.60 (d, 13.7, 1H)	1.84 (m, 1H), 2.63 (dd, 3.3, 18.9, 1H)
17	1.72 (s, 3H)	1.27 (s, 3H)	0.81 (s, 3H)
19	1.17 (s, 3H)	1.16 (s, 3H)	1.23 (s, 3H)
20	0.99 (s, 3H)	0.98 (s, 3H)	0.94 (s, 3H)
1′	4.66 (d, 7.8, 1H)	4.66 (d, 8.2, 1H)	5.39 (d, 8.2, 1H)
2′	3.57 (m, 1H)	3.56 (m, 1H)	3.33 (dd, 7.5, 8.0, 1H)
3′	3.77 (m, 1H)	3.74 (m, 1H)	3.43 (dd, 8.2, 8.9, 1H)
4′	3.36 (m, 1H)	3.34 (m, 1H)	3.32 (dd, 8.4, 9.2, 1H)
5′	3.28 (m, 1H)	3.30 (m, 1H)	3.36 (ddd, 8.4, 1.8, 6.9, 1H)
6′	3.64 (m, 1H), 3.80 (m, 1H)	3.67 (m, 1H), 3.88 (m, 1H)	3.67 (dd, 2.1, 11.4, 1H), 3.80 (dd, 4.2, 12.2, 1H)
1′′	4.85 (d, 7.8, 1H)	4.89 (d, 7.8, 1H)	
2′′	3.24 (m, 1H)	3.25 (m, 1H)	
3′′	3.32 (m, 1H)	3.42 (m, 1H)	
4′′	3.33 (m, 1H)	3.32 (m, 1H)	
5′′	3.33 (m, 1H)	3.36 (m, 1H)	
6′′	3.56 (m, 1H), 3.81 (m, 1H)	3.60 (m, 1H), 3.80 (m, 1H)	
1′′′	4.72 (d, 7.8, 1H)	4.74 (d, 7.8, 1H)	
2′′′	3.27 (m, 1H)	3.25 (m, 1H)	
3′′′	3.33 (m, 1H)	3.32 (m, 1H)	
4′′′	3.32 (m, 1H)	3.32 (m, 1H)	
5′′′	3.36 (m, 1H)	3.36 (m, 1H)	
6′′′	3.67 (m, 1H), 3.71 (m, 1H)	3.65 (m, 1H), 3.80 (m, 1H)	

**^a^** assignments made on the basis of COSY, HSQC and HMBC correlations; **^b^** Chemical shift values are in δ (ppm); **^c^** Coupling constants are in Hz.

**Table 2:** ^13^C NMR chemical shift values for **1**–**3** isolated from *Stevia rebaudiana* recorded in CD_3_OD ^a^.

Position	1	2	3
1	40.9	42.0	39.7
2	19.1	19.0	19.2
3	37.9	38.0	38.1
4	43.5	43.5	43.8
5	56.5	55.3	57.4
6	20.9	21.2	20.8
7	39.6	39.3	41.3
8	48.3	41.8	48.6
9	48.0	55.2	54.0
10	39.8	40.6	37.8
11	20.6	20.2	21.2
12	30.4	37.2	38.7
13	91.4	86.8	94.4
14	48.1	43.4	54.7
15	136.1	56.9	48.1
16	142.6	76.8	224.0
17	11.4	21.8	12.9
18	180.4	180.3	176.9
19	29.0	28.3	27.8
20	15.3	15.1	19.0
1′	96.6	95.8	95.3
2′	80.1	79.8	73.8
3′	87.5	86.8	77.4
4′	70.2	69.8	69.9
5′	77.3	77.2	77.5
6′	61.4	61.5	61.2
1′′	103.1	103.0	
2′′	74.2	74.0	
3′′	77.3	77.4	
4′′	70.3	70.2	
5′′	77.1	77.2	
6′′	61.4	61.3	
1′′′	102.5	102.6	
2′′′	73.8	74.6	
3′′′	77.1	77.0	
4′′′	70.3	70.4	
5′′′	77.4	77.2	
6′′′	61.5	61.3	

**^a^** assignments made on the basis of HSQC and HMBC correlations; **^b^** Chemical shift values are in δ (ppm).
